# Criminalizing Serious Mental Illness: What Linked Urban Data Reveal and the Case for Community Care

**DOI:** 10.1007/s11524-026-01101-2

**Published:** 2026-05-25

**Authors:** Michelle Suh, Justin Berk, Lauren Brinkley-Rubinstein, Josiah Rich

**Affiliations:** 1 Department of Emergency Medicine, Brown University Health, 55 Claverick St, Providence, RI 02903 USA; 2 Warren Alpert School of Medicine, Brown University, 222 Richmond St, Providence, RI 02903 USA; 3 Department of Medicine and Pediatrics, Warren Alpert Medical School at Brown University, 222 Richmond St, Providence, RI 02903 USA; 4 Bellwether Collaborative for Health Justice, Department of Population Health Sciences, Duke University School of Medicine, 215 Morris Street, Suite 210, Durham, NC 27701 USA; 5 Division of Infectious Diseases, Warren Alpert Medical School, Brown University, 222 Richmond St, Providence, RI 02903 USA; 6 The Center for Health and Justice Transformation, The Miriam Hospital, Providence, RI USA; 7 COBRE on Opioids and Overdose at Rhode Island Hospital, 1125 N Main St, Suite 215, Providence, RI 02906 USA; 8 Collaborative Justice-Involved Research and Training Program on Substance Use and HIV, Brown University Health, 167 Point St, Providence, RI 02903 USA

**Keywords:** Prison, Jail, Incarceration, Serious Mental Illness, Data Linkage, Emergency Department

## Abstract

The United States has responded to multiple societal ills, from substance use to homelessness to mental health, with criminalization. A new analysis using City and County of San Francisco data shows jails disproportionately harm people with serious mental illness (SMI). In addition to longer periods of incarceration and higher rates of repeat jail bookings, people with SMI also experience higher rates of homelessness, co-occurring substance use disorder, and urgent and emergent health services utilization before and after incarceration compared to their peers without SMI. The study provides a much-needed spotlight on jail research, as well as demonstrating the power of data when not siloed in multiple, disparate departments. Investing in community-based mental health resources, housing, and substance use disorder services can address the upstream determinants of incarceration and may benefit people with SMI.

The United States has the largest incarcerated population in the world, with almost 2 million people in jails and prisons and over 3 million people on probation and parole. This reflects the country’s cultural response to societal ills primarily through incarceration. For example, the primary response to substance use has been the “War on Drugs”, marked by more punitive laws resulting in an increase in drug-related arrests despite research showing that harsher prison terms have not deterred drug misuse, distribution, and recidivism [1–4]. Even now, the United States is experiencing an opioid crisis with significant morbidity and mortality marked by high rates of incarceration [5, 6]. Similarly, homelessness is on the rise in the United States, primarily due to lack of investment in affordable housing and increasing cost of living. However, multiple cities, counties, and states have responded to the housing crisis by criminalizing homelessness. Examples include restricting feeding people experiencing homelessness, [7] “nuisance laws”, [8] and most recently, the United States Supreme Court case Grants Pass v. Johnson which allows cities to ban people from sleeping and camping in public spaces. [9] Similarly, despite high rates of depression, schizophrenia and other serious mental illnesses (SMI) in our country, [10] psychiatric symptoms are too often met with criminalization. [11] After deinstitutionalization occurred in the 1980s with the promise of safety net community care systems that never materialized, correctional facilities have become the de-facto institutions to address SMI. However, incarceration itself often causes psychological harm and worsening mental illness. [12] Hewlett et al. extends this work to demonstrate that jails disproportionately harm people with SMI.

This retrospective cross-sectional study utilized local health services and jail system data from the City and County of San Francisco to compare health services use, jail system incarceration, and associated factors among individuals with a jail incarceration stratified by the presence or absence of an SMI diagnosis. Individuals with SMI had higher rates of substance use disorder and homelessness than individuals without SMI. In addition to higher rates of jail reincarceration, individuals with SMI also had longer jail incarceration stays than individuals without SMI.

This study is notable for several reasons. First, the majority of carceral health research focuses on prisons. However, the jail setting is not the same and deserves its own research to identify its unique patterns and potential opportunities for intervention. For example, the jail population is highly transient compared to the prison population. There is a more than 20-fold greater number of people passing through jails than prisons. As a result, as demonstrated by Hewlett et al., close coordination with community partners, such as local emergency departments, represents a powerful opportunity for meaningful intervention that has the potential to reach millions of people annually.

Second, the study’s analysis is effective in part due to the extraordinary data collection and access. Specifically, the San Francisco Department of Public Health’s (SFDPH) Coordinated Care’s Management System (CCMS) and county jail system data from San Francisco Sheriff’s office not only collect comprehensive data but also are accessible to researchers. This allows for evidence-based problem identification; focus on specific populations; and development, implementation, and evaluation of public health interventions. More thoughtful data collection and coordination across government agencies may give rise to further research insights. Additionally, at a time of great excitement over the possibility of artificial intelligence (AI), these large datasets may be an opportunity to deploy AI for analysis. This should be a model that could be explored in many if not most jurisdictions.

Third, the study shows individuals with SMI have higher rates of emergency department (ED) utilization as well as mental health services visits. Nationally, EDs are experiencing high patient volumes with an increase in crowding and boarding.[13, 14] Individuals with SMI and a history of incarceration may be a potential population to offer targeted interventions. These interventions should provide alternate options, such as same-day clinic appointments and primary care services, and may help reduce the burden on the ED while ensuring these patients receive appropriate care.

The authors highlight the systemic barriers for individuals with SMI and call for addressing the upstream drivers of incarceration. The authors emphasize the need for health policies that prioritize investment in community-based mental health resources, housing, and substance disorder services. We echo these calls to address inadequate care coordination, geographic disparities and financial barriers to mental health services; increased supportive and affordable housing; and community-based diversion programs.

At the same time, improving the services at jails can directly benefit the population currently incarcerated. This may include providing mental health services such as screening at intake, routine check-ins, and hiring and training mental health staff; initiating MOUD; and more robust re-entry programming, such as transition clinics, linkages to care, and close coordination with community partners.

These solutions are not novel, and they have been successfully implemented elsewhere. As Hewlett et al. highlight, other countries have successfully implemented Housing First programs that demonstrated a reduction in homelessness and improvement in mental health outcomes.[15, 16] Within the United States, alternative crisis response models, such as a mobile crisis team, have also provided health and social services without placing individuals with SMI back into the carceral apparatus [17, 18].

Fundamentally, we challenge the framework of mass incarceration as a productive response to society’s ills. The United States spends over $100 billion on prisons and jails alone, a larger proportion of its population than any other country. [19] In addition to the financial burden of mass incarceration, the United States also pays the price in terms of the negative impacts of incarceration on health for those incarcerated, as well as families and communities affected. This situation is an indictment of our country’s overreliance on incarceration as well as underfunding of community mental health resources and other supports for people with SMI. As Hewlett et al. have shown in their study, this is not an effective approach. We can start to improve this system by ensuring that those currently caught up in it with SMI are better treated and, ideally, never sent there in the first place.
